# Psycho-Educational Assessments: Theory and Practice

**DOI:** 10.3390/jintelligence12030031

**Published:** 2024-03-05

**Authors:** Okan Bulut

**Affiliations:** Centre for Research in Applied Measurement and Evaluation, Faculty of Education, University of Alberta, Edmonton, AB T6G 2G5, Canada; bulut@ualberta.ca

Psycho-educational assessments, such as intelligence tests, cognitive test batteries, and behavioral measures, serve as invaluable tools for school psychologists and educators. They provide profound insights into children’s learning and behavioral profiles, enhancing our understanding of their academic and cognitive capacities. By employing these assessments, professionals can pinpoint each student’s individual strengths and weaknesses. Moreover, psycho-educational assessments play a pivotal role in identifying various educational needs, including learning disabilities, intellectual differences, social-emotional challenges, and giftedness. These assessments offer a comprehensive view of students’ abilities and potential hurdles they may encounter in their academic journey. Beyond diagnosis, the results of psycho-educational assessments can also inform the development of tailored interventions and support programs. They enable educators to implement timely strategies that address the unique requirements of students, fostering an inclusive learning environment where every child can thrive.

This Special Issue on “Psycho-Educational Assessments: Theory and Practice,” presented by the Journal of Intelligence, provided us with the opportunity to curate a collection of sixteen articles that delve into different facets of psycho-educational assessments. Each article serves as a testament to our remarkable journey in understanding and enhancing the psycho-educational assessment landscape. For this Special Issue, researchers were asked to present findings of empirical or methodological research and theoretical work related to the design, use, analysis, interpretation, and reporting processes of psycho-educational assessments. All articles submitted for publication underwent a rigorous peer review based on the review standards established by the Journal of Intelligence.

For the sake of brevity, I want to touch upon the focal points of this Special Issue and highlight key articles. The authors dedicated their efforts to exploring the identification of cognitive, social, emotional, and behavioral challenges across diverse groups, ranging from children aged 6 to 11, individuals with autism spectrum disorder, and verbally gifted children to those from regions like China and the United Arab Emirates. These studies primarily relied on psycho-educational assessments, employing intelligence tests, behavioral checklists, and psychological scales as primary data collection tools. Notably, intelligence tests, given their increasing significance in educational contexts, featured prominently in this Special Issue. Several studies leveraged well-established cognitive assessment batteries, including the Wechsler Intelligence Scale for Children, the Wechsler Individual Achievement Test, and the Woodcock–Johnson Tests of Academic Achievement and Cognitive Abilities. In addition to empirical investigations, the Special Issue encompassed methodological inquiries. For instance, Gao et al. (2022) conducted an empirical study focusing on process data indicators to explore problem-solving styles within technology-rich environments. Furthermore, Meyer and Reynolds (2022) demonstrated a methodological approach, multidimensional scaling, to scrutinize correlations between cognitive abilities and academic achievement test scores.

As the editor of this special issue, I anticipate that the articles showcased herein will serve as catalysts for meaningful discourse, igniting innovative ideas and enriching the ongoing evolution of psycho-educational assessment. Moreover, these featured articles will provide valuable insights into enhancing various aspects of the design, utilization, analysis, interpretation, and reporting processes involved in psycho-educational assessments. The contributions within this collection delve into diverse facets, from exploring the efficacy of psycho-educational assessments in diagnosing a range of learning difficulties to different statistical techniques for analyzing psycho-educational constructs, such as local structural equation models and multidimensional scaling.

In closing this editorial summary, I wish to express my heartfelt gratitude to the esteemed authors whose expertise and dedication have profoundly enriched this Special Issue. A special acknowledgment extends to all the diligent reviewers who generously shared their valuable insights, contributing significantly to the refinement of the submitted papers. Additionally, I express my sincere appreciation to the Editorial Team of the Journal of Intelligence for entrusting me with the privilege of curating this Special Issue. This Special Issue would not have been possible without their unwavering support and meticulous efforts.
